# Severe traumatic atlantoaxial dislocation and type III odontoid fracture treated with occipitocervical fixation: a case report

**DOI:** 10.1093/jscr/rjae281

**Published:** 2024-05-02

**Authors:** Tjokorda Gde Bagus Mahadewa, Eufrata Silvestris Junus, Steven Awyono, Denny Japardi, Christopher Lauren

**Affiliations:** Neurosurgery Division, Department of Surgery, Faculty of Medicine, Universitas Udayana, Prof. Dr. I.G.N.G. Ngoerah General Hospital, Denpasar, Bali, Indonesia; Neurosurgery Division, Department of Surgery, Faculty of Medicine, Universitas Udayana, Prof. Dr. I.G.N.G. Ngoerah General Hospital, Denpasar, Bali, Indonesia; Neurosurgery Division, Department of Surgery, Faculty of Medicine, Universitas Udayana, Prof. Dr. I.G.N.G. Ngoerah General Hospital, Denpasar, Bali, Indonesia; Neurosurgery Division, Department of Surgery, Faculty of Medicine, Universitas Udayana, Prof. Dr. I.G.N.G. Ngoerah General Hospital, Denpasar, Bali, Indonesia; Neurosurgery Division, Department of Surgery, Faculty of Medicine, Universitas Udayana, Prof. Dr. I.G.N.G. Ngoerah General Hospital, Denpasar, Bali, Indonesia

**Keywords:** atlantoaxial dislocation, cervical spine, neurosurgery, odontoid fracture, occipitocervical fixation

## Abstract

The combination of atlantoaxial joint dislocation accompanied by an odontoid process fracture is exceptionally rare, with only a few cases reported. The estimated frequency of these cases is < 2% of all upper cervical spine injuries. In this report, the authors describe an unusual case of traumatic atlantoaxial dislocation with a type III odontoid fracture in a 44-year-old male patient. Before the diagnosis, the patient had a history of seeking a masseur for a neck massage. Subsequently, the patient underwent occipitocervical stabilization to address the underlying condition. This procedure aims to treat the instability between the skull and cervical spine and should be considered in the treatment planning if the patient’s anatomy suits it.

## Introduction

Traumatic atlantoaxial dislocation constitutes 2.7% of all cervical injury cases [1]. Even rarer are cases of atlantoaxial dislocation (AAD) accompanied by odontoid fractures, with only a total of eight such cases previously reported [2–8]. The estimated frequency of these occurrences is < 2% of all upper cervical spine injuries, and among these cases, only two were associated with type 3 odontoid fractures [9]. Occipitocervical fixation is indicated as a management approach for craniocervical instability, such as atlantoaxial dislocation and odontoid fracture. This procedure is technically challenging and complex. Currently, rigid occipital plating with bicortical screws connected using rods appears to be the preferred method for achieving proper and secure stabilization [10, 11].

We present the case of a 44-year-old male patient who had atlantoaxial dislocation accompanied by a type III odontoid fracture. In this report, we provide a comprehensive account of our experience with this patient, including their medical history, physical examination, diagnostic support, and the surgical procedure performed, with reference relevant literature for context.

## Case report

A 44-year-old male patient arrived at the emergency department, reporting tingling sensations in his hands and feet persisting for the past week. This discomfort began after he received a neck massage at a local massage parlor. Initially, the patient had experienced soreness in the neck region and decided to undergo a massage to alleviate the discomfort. The following day, the patient started to feel pain in the neck area, along with numbness in all four extremities. Notably, there were no issues with urination or defecation.

**Figure 1:**
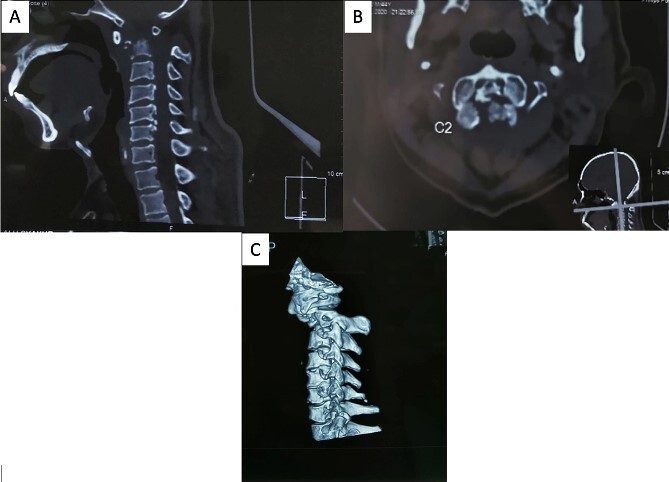
A cervical CT scan revealed an odontoid fracture and severe atlantoaxial dislocation. (A) Sagittal view, (B) axial view, and (C) lateral view of three-dimensional CT revealed atlantoaxial dislocation with odontoid fracture.

**Figure 2:**
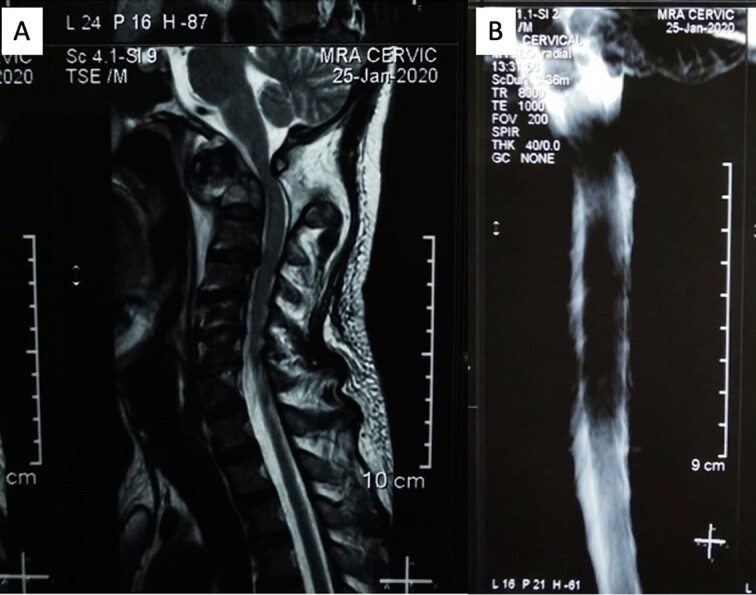
(A) Sagittal MRI showed significant compression on the occipitocervical junction due to atlantoaxial dislocation with blockage from (B) MR myelography.

The patient reported sensory disturbances in all four extremities, particularly related to abnormalities in light touch, without any other sensory qualities being affected. A Cervical CT scan revealed a type III odontoid fracture, D’Alonzo type III, with atlantoaxial dislocation, fielding grade 3, and ossification of the posterior longitudinal ligament on the C4-C6 levels (Fig. 1). Cervical MR imaging showed cervical canal stenosis at the C1-C2 level due to atlantoaxial dislocation (Fig. 2). Consequently, we planned to proceed with stabilization surgery. We were unable to perform angiography on this patient due to limited facility resources for such examinations.

## Surgical procedure

**Figure 3:**
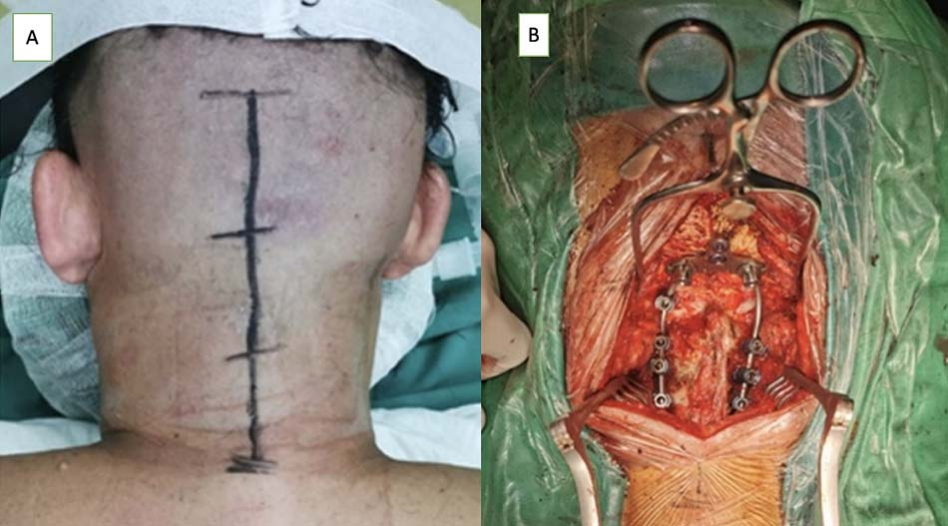
(A) Midline skin incision was marked to expose from occipital bone to C3 level and (B) occiptocervical fixation using an occipital plate that fixated on occipital squama connected by a surgical rod through the C4 on both sides.

Before the operative procedure was performed, the patient was immobilized using a Philadelphia collar. The patient was then induced under general anesthesia, with fiberoptic intubation used for intubation. We shaved and sterilized the craniocervical region using an antiseptic agent and ensured proper draping. A midline skin incision was made subperiosteally, exposing the area from the inion to the C3 region, with a width of ~6 cm (Fig. 3A). Special attention was given when dissecting around the C1 region to avoid potential vascular injuries. Drilling was performed on the occipital bone to create a flat surface, and a high-speed drill was used to insert an occipital screw with a length of ~10 mm. Lateral mass screws were utilized for cervical instrumentation, and the size of the midline occipital plate was chosen to align with the rod attachment points, which were then fixed with the lateral mass screws (Fig. 3B). During the screw placement process, we used a C-arm and our anatomical knowledge to ensure proper screw trajectory. We did not utilize electrophysiological monitoring during the operative procedure as we did not have that facility in our institution. Bone graft was not used in this patient. Wound closure was meticulously performed layer by layer, with careful reconstruction of the paraspinal muscles. Following the operative procedure, a soft collar was applied postoperatively to the patient’s neck.

**Figure 4:**
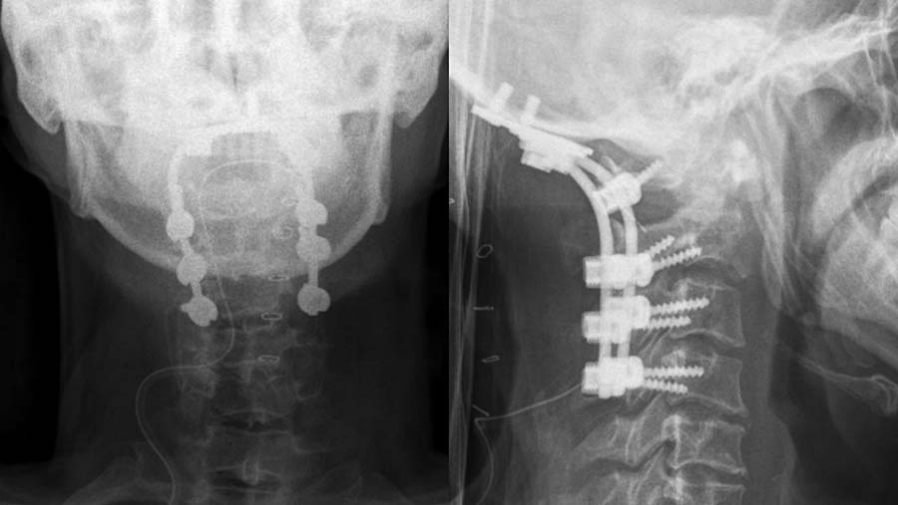
Postoperative x-ray showed proper occipitocervical stabilization.

Following the surgery, the patient remained an inpatient for 5 days and was subsequently discharged from our ward. The patient reported a reduction in tingling sensations in both hands and feet. A postoperative X-ray confirmed the successful occipitocervical fusion with a well-maintained screw trajectory (Fig. 4).

## Discussion

Traumatic atlantoaxial dislocation accounts for 2.7% of all cases of cervical injuries [1]. This condition arises from external forces that disrupt the C1 transverse ligament. However, the combination of atlantoaxial joint dislocation accompanied by an odontoid process fracture is exceptionally rare, with only eight cases reported to date (Table 1) [2–8]. The estimated frequency of these cases is <2% of all upper cervical spine injuries [9]. Among the eight reported cases, the combination of injuries always involved a C1–C2 Type IV dislocation based on the Fielding classification, accompanied by a type II odontoid fracture in six cases, or it could also be accompanied by a type III odontoid fracture in two cases, according to the Anderson and D’Alonzo classification. In contrast to the cases previously reported, our case is unique. There has never been a previous case report that described a combination of injuries involving a C1–C2 dislocation Type III according to the Fielding classification and a type III odontoid fracture based on the Anderson and D’Alonzo classification.

**Table 1:** Reported cases of combined traumatic atlantoaxial dislocation with odontoid fracture

	**Authors**	**Year**	**Age**	**Sex**	**Classification**		**Treatment**
					**Anderson**	**Fielding**	
1	Autrique *et al.*	1986	45 years	M	Type II	Type IV	Halo-vest
2	Autrique *et al.*	1986	63 years	M	Type II	Not specified	Posterior occipito - C3 fusion with iliac graft
3	Fuentes *et al.*	2001	24 years	M	Type II	Type IV	Posterior C1 – C2 fusion
4	Spoor *et al.*	2008	43 years	M	Type II	Not specified	Halo-vest
5	Lenehan *et al.*	2009	63 years	F	Type II	Not specified	Open reduction, posterior transarticular fusion
6	Hopf *et al.*	2009	15 years	F	Type II	Type IV	Posterior transarticular fusion, bicortical iliac crest graft
7	Oh *et al.*	2010	37 years	M	Type III	Not specified	Halo-vest
8	Moreau *et al.*	2012	65 years	M	Type II	Type IV	Open reduction, occipitocervical fixation
9	Present case	2023	44 years	M	Type III	Type III	Open reduction, occipitocervical fixation

M, Male; F, Female

Atlantoaxial dislocation disrupts the physiological articulation of C1–C2, leading to instability [12]. Neck pain typically serves as the initial symptom of an odontoid fracture, and sometimes neurological deficits may occur, although this is rare [13]. In this case, the patient initially experienced neck pain after receiving a massage on the neck in a massage parlor. There is a possibility that the massage triggered the atlantoaxial joint dislocation, subsequently leading to odontoid fractures. The tingling sensations in the patient’s hands and feet may also indicate that the disruption of ligaments in the atlantoaxial joint resulted in neurological damage.

Radiographic measurement of the atlantoaxial joint articulation defines atlantoaxial dislocation by assessing the atlantodental interval [14]. Fielding and Hawking classified atlantoaxial dislocations into four categories. Type I involves the odontoid process acting as a rotational center without any displacement in the axial plane. Type II is characterized by the rotational center being lateral to the C1–C2 joint. Type III entails bilateral atlantoaxial joint subluxation with both rotational and anterior deviation exceeding 5 mm, and Type IV involves a posterior subluxation of both atlantoaxial joints [12, 13]. In this specific case, the patient has been diagnosed with a type III atlantoaxial dislocation according to the fielding classification, determined through radiographic examination.

Atlantoaxial dislocation with type II and III odontoid fractures resulting from trauma typically necessitates surgical treatment for posterior stabilization [15]. However, surgical reduction and fixation of atlantoaxial dislocation come with inherent risks, one of the most serious being vertebral artery injury, with reported incidences ranging from 0% to 8.2% for posterior atlantoaxial transarticular screw fixation. In this case, the patient is scheduled for occipitocervical fixation surgery, extending from the occipital bone through the fourth cervical fusion. Stabilization in such cases is particularly challenging due to the highly dynamic interaction between the occipital condyles and the atlantoaxial joints, often associated with a complex force vector between the skull and the spine [10].
